# Supplementary material for: Hepatitis Risk in Diabetes Compared to Non-Diabetes and Relevant Factors: A Cross-Sectional Study with National Health and Nutrition Examination Survey (NHANES), 2013–2018
Source: Int J Environ Res Public Health. 2023 Mar 11;20(6):4962. doi: 10.3390/ijerph20064962 (PMC10049568; doi:10.3390/ijerph20064962)
Supplement: Supplementary file 1 [file ijerph-20-04962-s001.zip › ijerph-2240646-supplementary.pdf]

Supplementary Material Table S1. Variables compare between including vs. excluding data with illegal drug use

**Variables**	**All without response to illicit drug (2013-2018)**	**All with response to illicit drug (2013-2018)**
**Age (N)**		
0~19	696(6.00%)	6,694(31.73%)
20~44	5421(46.73%)	6,033(28.60%)
45~64	4,506(38.84%)	4,992(23.67%)
≥65	978(8.43%)	3,375(16.00%)
**Sex (N)**		
Female	5,986(51.60%)	10,810(51.25%)
Male	5,615(48.40%)	10,284(48.75%)
**BMI (N)**		
Underweight (<18.5kg/m²)	199(1.72%)	2,634(12.49%)
Normal (18.5~24.9kg/m²)	3,220(27.76%)	6,577(31.18%)
Obese (≥25kg/m²)	8,182(70.53%)	11,883(56.33%)
**Race (N)**		
Mexican American	1,839(15.85%)	3,388(16.06%)
Other Hispanic	1,213(10.46%)	2,114(10.02%)
Non-Hispanic White	4,114(35.46%)	7,446(35.30%)
Non-Hispanic Black	2,550(21.98%)	4,649(22.04%)
Non-Hispanic Asian	1,368(11.79%)	2,430(11.52%)
Other Race - Including Multi-Racial	517(4.46%)	1,067(5.06%)
**Poverty (N)**		
Yes	2,587(22.30%)	5,145(24.39%)
No	9,014(77.70%)	15,949(75.61%)
**Hepatitis B (N)**		

Yes	172(1.48%)	238(1.37%)
No	11,429(97.91%)	20,856(98.63%)
**Hepatitis C (N)**		
Yes	243(2.09%)	289(1.37%)
No	11,358(97.91%)	20,805(98.63%)
**Hepatitis B or C (N)**		
Yes	389(3.35%)	495(2.35%)
No	11,212(96.65%)	20,599(97.65%)
**Illicit Drug (N)**		
Yes	285(2.46%)	285(2.46%)
No	11,316(97.54%)	11,316(97.54%)

Supplementary Material Table S2. Association of diabetes mellitus status with hepatitis group.

**Variables**	**Hepatitis B**				**Hepatitis C**				**Hepatitis B or C**			
	**OR**	**95% CI**		**P**	**OR**	**95% CI**		**P**	**OR**	**95% CI**		**P**
		**lower**	**upper**			**lower**	**upper**			**lower**	**upper**	
Non-DM	REF				REF				REF			
DM	1.772	1.239	2.534	0.002	1.675	1.232	2.277	0.001	1.730	1.356	2.206	<0.0001
**Stratified by age**												
0~19												
Non-DM	REF				REF				REF			
DM	0.000	0.000	INF	0.997	1.000	0.000	INF	1.000	0.000	0.000	INF	0.997
20~44												
Non-DM	REF				REF				REF			
DM	1.934	0.824	4.538	0.130	1.856	0.656	5.247	0.244	1.577	0.757	3.287	0.224
45~64												
Non-DM	REF				REF				REF			
DM	1.242	0.780	1.978	0.362	0.930	0.638	1.356	0.706	1.086	0.803	1.471	0.592
≥65												
Non-DM	REF				REF				REF			
DM	1.338	0.566	3.063	0.507	0.995	0.515	1.924	0.988	1.095	0.630	1.903	0.747
**Stratified by sex**												
Male												
Non-DM	REF				REF				REF			

DM	1.697	1.076	2.677	0.023	1.582	1.078	2.323	0.019	1.686	1.242	2.290	0.001
Female												
Non-DM	REF				REF				REF			
DM	1.803	1.011	3.213	0.046	1.732	1.037	2.891	0.036	1.701	1.136	2.544	0.010
**Stratified by BMI**												
Underweight (<18.5)												
Non-DM	REF				REF				REF			
DM	0.000	0.000	INF	0.995	0.000	0.000	INF	0.995	0.000	0.000	INF	0.995
Normal (18.5~24.9)												
Non-DM	REF				REF				REF			
DM	2.455	1.150	5.240	0.020	1.541	0.699	3.393	0.283	1.934	1.089	3.435	0.024
Overweight (≥ 25)												
Non-DM	REF				REF				REF			
DM	1.853	1.222	2.809	0.004	1.863	1.320	2.630	0.000	1.889	1.414	2.493	<0.0001
**Stratified by race**												
Mexican American												
Non-DM	REF				REF				REF			
DM	0.669	0.150	2.977	0.598	1.384	0.549	3.492	0.491	0.953	0.417	2.178	0.909
Other Hispanic												
Non-DM	REF				REF				REF			
DM	1.985	0.699	5.630	0.198	0.532	0.123	2.301	0.398	1.056	0.433	2.578	0.905
Non-Hispanic White												
Non-DM	REF				REF				REF			
DM	2.412	1.782	4.939	0.016	1.713	1.032	2.842	0.037	1.961	1.272	3.023	0.002
Non-Hispanic Black												
Non-DM	REF				REF				REF			
DM	1.579	0.705	3.537	0.267	2.183	1.319	3.610	0.002	2.059	1.327	3.196	0.001
Non-Hispanic Asian												
Non-DM	REF				REF				REF			
DM	2.337	1.252	4.362	0.008	0.000	0.000	INF	0.994	2.182	1.174	4.054	0.014
Other Race - Including												

Multi-Racial												
Non-DM	REF				REF				REF			
DM	1.031	0.122	8.687	0.978	2.850	0.586	9.510	0.089	1.811	0.579	5.664	0.307
**Stratified by poverty**												
Yes												
Non-DM	REF				REF				REF			
DM	1.389	0.709	2.721	0.338	1.554	0.974	2.479	0.064	1.555	1.040	2.325	0.032
No												
Non-DM	REF				REF				REF			
DM	1.940	1.271	2.961	0.002	1.702	1.130	2.563	0.011	1.795	1.321	2.439	0.002
**Stratified by illicit drug use**												
Yes												
Non-DM	REF				REF				REF			
DM	1.145	0.368	3.564	0.815	2.027	1.089	3.774	0.026	1.979	1.062	3.689	0.032
No												
Non-DM	REF				REF				REF			
DM	1.839	1.260	2.684	0.002	1.580	1.030	2.425	0.036	1.720	1.284	2.304	<0.001

Note: CI: Confidence Interval; DM: Diabetes Mellitus; OR: Odds Ratio; P: P-value; REF: Refere

Supplementary Material Table S3. Prevalence of hepatitis B or C with in the diabetes mellitus population according to FPG and HbA1c levels.

Variables	Participants (N)	Univariate Analysis			
		OR	95% CI		P
			lower	upper	
**HbA1c**					
≥9.0	303	REF			
<9.0	1,376	1.023	0.995	1.052	0.103
**FPG**					
≥200	189	REF			
<200	694	1.023	0.984	1.063	0.249

CI: Confidence Interval; OR: Odds Ratio; P: P-value; FPG: Fasting Plasma Glucose; HbA1c: Hemoglobin A1c; REF: Reference.

Supplementary Material Figure S1. Prevalence of hepatitis regarding the fast glucose or glycated hemoglobin levels.

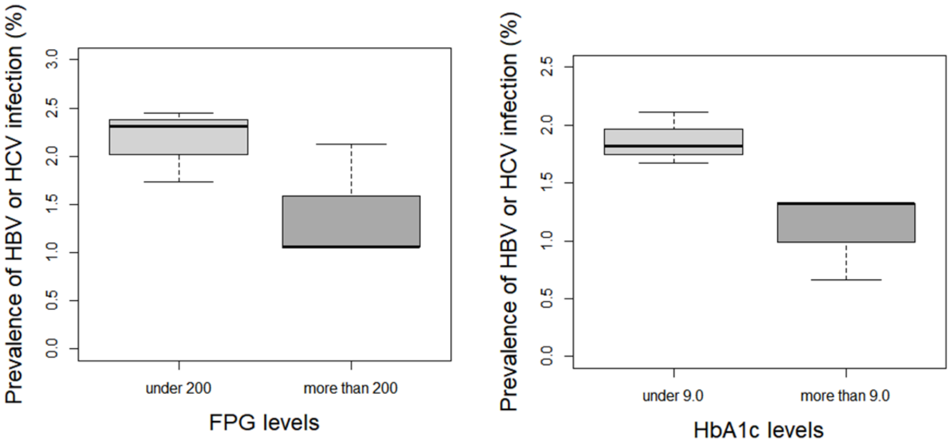

Prevalence of HBV or HCV infection (%)
0.0
0.5
1.0
1.5
2.0
2.5
3.0
under 200
more than 200
FPG levels
Prevalence of HBV or HCV infection (%)
0.0
0.5
1.0
1.5
2.0
2.5
under 9.0
more than 9.0
HbA1c levels

Supplementary Material Figure S2. Prevalence of each hepatitis by the 2-year-cycle according to diabetes mellitus status from 2013 to 2018.

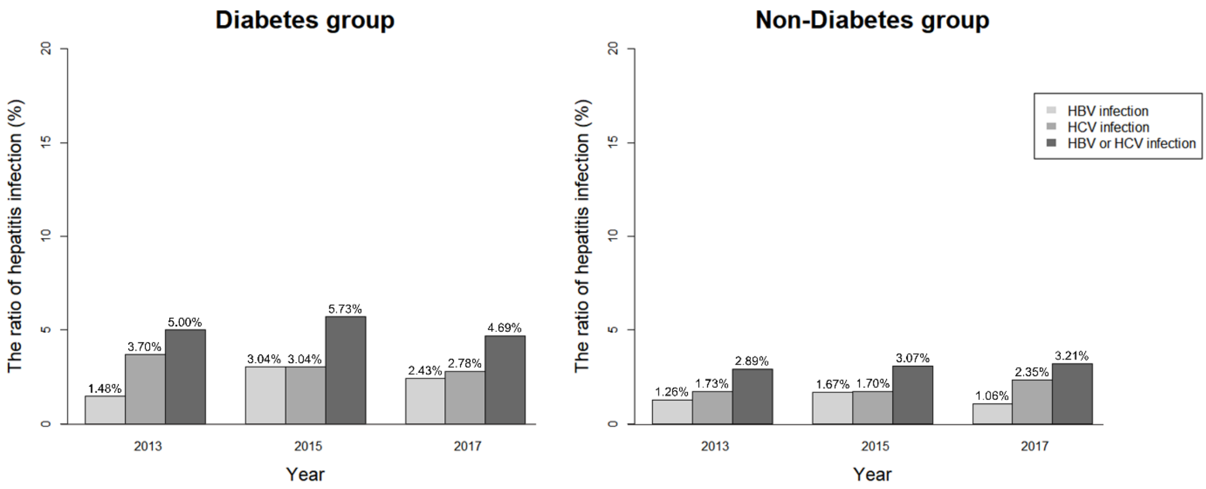

Note: DM: Diabetes Mellitus; HBV: Hepatitis B virus; HCV: Hepatitis C virus.
